# Practical parameter identifiability and handling of censored data with Bayesian inference in mathematical tumour models

**DOI:** 10.1038/s41540-024-00409-6

**Published:** 2024-08-14

**Authors:** Jamie Porthiyas, Daniel Nussey, Catherine A. A. Beauchemin, Donald C. Warren, Christian Quirouette, Kathleen P. Wilkie

**Affiliations:** 1 Department of Mathematics, Toronto Metropolitan University, Toronto, ON M5B 2K3 Canada; 2 Department of Physics, Toronto Metropolitan University, Toronto, ON M5B 2K3 Canada; 3 Interdisciplinary Theoretical and Mathematical Sciences (iTHEMS) Program, RIKEN, Wako-shi, Saitama 351-0198 Japan; 4 Florida Institute of Technology, Melbourne, FL 32901 USA

**Keywords:** Applied mathematics, Computational science, Cancer

## Abstract

Mechanistic mathematical models (MMs) are a powerful tool to help us understand and predict the dynamics of tumour growth under various conditions. In this work, we use 5 MMs with an increasing number of parameters to explore how certain (often overlooked) decisions in estimating parameters from data of experimental tumour growth affect the outcome of the analysis. In particular, we propose a framework for including tumour volume measurements that fall outside the upper and lower limits of detection, which are normally discarded. We demonstrate how excluding censored data results in an overestimation of the initial tumour volume and the MM-predicted tumour volumes prior to the first measurements, and an underestimation of the carrying capacity and the MM-predicted tumour volumes beyond the latest measurable time points. We show in which way the choice of prior for the MM parameters can impact the posterior distributions, and illustrate that reporting the most likely parameters and their 95% credible interval can lead to confusing or misleading interpretations. We hope this work will encourage others to carefully consider choices made in parameter estimation and to adopt the approaches we put forward herein.

## Introduction

Mathematical models are the primary tools by which we can examine biological or clinical data to determine fundamental mechanisms, to test hypotheses, and to make predictions. A mechanistic mathematical model formalises assumptions of causality by describing underlying mechanisms with the aim of exploring the limits and consequences of the input-output relationship. Hereafter, the focus is on ordinary differential equation-based mechanistic models (MMs). In the process of formalising biological processes and functions into mathematical forms, parameter values are introduced. The values of these parameters then become key factors in the MM’s predictions and are typically estimated by fitting the MM to data (least-square approach) or estimating their likelihood given the observed data (Bayesian approach). Determining MM parameters given an observed outcome is a challenging inverse problem that is highly sensitive to noise.

MMs can be used to predict an average response (by using a set of parameter values based on all trajectories at once or on their average), to predict a specific response (by using a set of parameter values based on a specific trajectory in a dataset), and to predict population-level responses (by using an ensemble of parameter value sets obtained in a manner that generates parameter joint-distributions). Prediction of a patient-specific response requires estimating based on a specific trajectory and can be used to develop a digital twin if the estimation is repeated to update parameter values as new data is obtained^1^. Population responses require the MM to be extended by a virtual clinical trial where the response is tracked for a virtual population represented by an ensemble of parameter value sets^2^.

Model identifiability examines whether or not the parameters can be uniquely determined given the system inputs and outputs^3,4^. A MM is classified as structurally identifiable if model parameters can be determined uniquely via the model structure under the assumption that the outputs are error-free. If additionally, model parameters are uniquely determined even in the presence of noise in the outputs, then the model is said to be practically identifiable^3^. Simply put, if two different sets of parameters exist that give identical dynamics, then the MM is not structurally identifiable, and will implicitly also not be practically identifiable. A MM can fail to be practically identifiable for three main reasons: (1) the data is not sufficient to identify all model parameters (e.g., estimating the parameters of a logistic growth curve based on data that only demonstrates an exponential growth phase); (2) the MM is mis-specified, making one or more incorrect assumptions about the system or its measurements; or (3) two or more model parameters are somehow dependent or their actions are coupled as measured by the MM output (e.g., estimating the parameters of an exponential growth model with a growth rate *a* and death rate *b*, wherein only the net growth rate (*a* − *b*) is identifiable). In modelling applications, it is important to check for practical identifiability since model outputs are compared to noisy real-world data^4^, as is the case in the present work. For MM predictions to be well constrained beyond the data, the MM taken together with the dataset should be practically identifiable. Ways to improve identifiability generally include reducing the number of model parameters, collecting and using more data points or over an extended range, or additional input conditions such as varying the dose of a particular treatment or the initial conditions of the experiment.

In practical applications, notably in Bayesian parameter estimation, a greater level of noise in the data translates to wider, more poorly constrained parameter distributions. But this so-called noise is typically the combined result of (1) experimental measurement accuracy (signal-to-noise ratio), which correspondingly degrades the accuracy of parameter estimates; and (2) inter-experimental variability such as that observed across a set of patients. If the latter is the dominant source of variability, which ideally should be the case, then the wider parameter distribution estimated when the data is particularly noisy could actually reflect the clinically relevant diversity of patient disease courses and outcomes. Rather than focusing on the best fit or mode or median parameter set, the whole of the posterior distribution can be used to sample parameter sets and simulate the heterogeneity of inter-patient response dynamics under a virtual clinical trial^5,6^. The posterior distribution forms an ensemble that can represent a virtual cohort, and the differing response dynamics then extend the capabilities of the model to explore potential heterogeneity in responses^2^.

For example, biological factors such as the sensitivity to angiogenic signals and the timescale associated with the sprouting and development of new blood vessels can affect the dynamic carrying capacity of a tumour microenvironment. This carrying capacity, modelled as either a constant parameter or a dependent state variable, plays a significant role in determining the dynamic behaviour of a growing tumour and its responsiveness to treatment^5–7^. Unfortunately, such factors are inherently difficult to estimate from data as no measurement can be taken directly on the capacity of a microenvironment to support a tumour. Therefore, in most oncology settings where data is limited, the parameter(s) associated with a carrying capacity are likely practically non-identifiable — but still incredibly useful as a mechanistic parameter or variable in the model^8^. In a logistic- or Gompertz-type MM, the ratio of tumour volume to carrying capacity slows down exponential growth until the maximum size is obtained. In the generalised logistic MM, there is an additional parameter that controls the strength of this ratio’s effect on tumour growth. The tumour volume to carrying capacity ratio is so significant, it was coined the proliferation saturation index in radiation response modelling^9^, and was shown to play a significant role in determining patient-specific responses to radiation in a MM where the radiation effect directly altered the carrying capacity^10^.

The best situation to parameterise a MM is to have complete time-series datasets for every output in the MM as well as additional insights or data on mechanisms described by the MM. Then, the MM can be parameterised and validated fully before being used to explore alternative situations from those described by the data^11^. If the aim is to study competing hypotheses for cancer development and treatment using MMs, then it is important that the parameters and/or the MM-predicted tumour growth curve beyond the extent of the data be sufficiently constrained by their estimation from available data, in order to challenge and discriminate between competing hypotheses. If a MM and dataset taken together are identifiable, then the region of highest likelihood in the parameter space will be well constrained and the resulting MM predictions will be as well.

On the other hand, increasingly complex MMs that capture the biological processes in greater detail, perhaps in order to correctly capture the mechanism behind a particular therapy, will lead to more parameters. This increase will likely ensure that the MM and data together are not identifiable, making the higher-dimensional parameter landscape more complex, with potentially many local minima, or disconnected parameter space regions of equivalently high likelihood. In such cases, a richer data set is required to adequately constrain the values of the additional MM parameters, although such data might not be available or even obtainable. Within the poorly constrained high likelihood regions of the parameter space, MM predictions could differ significantly and affect conclusions, if not realised and handled with care. In some cases, however, even poorly constrained parameters could still yield relatively well-constrained predictive time courses (e.g., tumour volumes beyond the measurement time points, or predictions of time courses under a simulated treatment regimen), which are sometimes more clinically useful and relevant than the parameters^12,13^.

Here, we consider several MMs of tumour growth with increasing complexity, and thus, increasing number of parameters. We use Bayesian inference to examine the ability of experimental data to constrain each MM’s parameters and explore the ability of the resulting parameterisations to predict growth beyond the measured time points. Specifically, we investigate the effects of including rather than neglecting data known to fall beyond the measurements’ limits of detection (censored data) and the choice of prior on the MM parameterisation results. Importantly, we show that neglecting the censored data leads to an underestimation of the tumour volume at early times and an overestimation at late times, resulting in an overestimation of the tumour’s age and an underestimation of the carrying capacity, two clinically meaningful quantities. Further, we demonstrate how the choice of prior can significantly alter parameter estimation, especially in MMs with more parameters, when the data is insufficient to adequately constrain all of them.

## Methods

### Considering a range of tumour growth MMs

Let us first consider a modified, special case of the generalised logistic growth equation, also known as Richards’ curve^14^

Rich MM $$\frac{{\rm{d}}C}{{\rm{d}}t}=\frac{\mu }{\min (\alpha ,1)}\,C\,\left[1-{\left(\frac{C}{\kappa }\right)}^{\alpha }\right]\quad C(t)=\frac{\kappa }{{\left[1+\left\{{\left(\kappa /{C}_{0}\right)}^{\alpha }-1\right\}{{\rm{e}}}^{-\max (1,\alpha )\mu t}\right]}^{(1/\alpha )}}$$

where *C*(*t*) is the tumour volume in mm^3^, and *κ* is both the fixed carrying capacity and the steady state of *C*(*t* → + *∞*). The coefficient $\mu /\min (\alpha ,1)$ is either *μ* for *α* ≥ 1 or *μ*/*α* for *α* < 1. This seemingly peculiar choice of coefficient better handles the change in the behaviour of this function about *α* = 1.

The Rich MM simplifies to the Logistic growth equation^15^ for *α* = 1,

Logis MM $$\frac{{\rm{d}}C}{{\rm{d}}t}=\mu \,C\,\left[1-\frac{C}{\kappa }\right]\quad C(t)=\frac{\kappa }{1+\left[\kappa /{C}_{0}-1\right]{{\rm{e}}}^{-\mu t}},$$

to the Gompertz growth equation^16^ as *α* → 0,

Gomp MM $$\frac{{\rm{d}}C}{{\rm{d}}t}=-\mu \,C\,\ln \left[\frac{C}{\kappa }\right]\quad C(t)=\kappa {\left[\frac{{C}_{0}}{\kappa }\right]}^{{{\rm{e}}}^{-\mu t}},$$

and to exponential growth capped at *κ* as *α* → + *∞*, namely

ExpCap MM $$C(t)\,\approx \min ({C}_{0}{{\rm{e}}}^{\mu t},\kappa ).$$

As the simplest MM we consider unbounded, exponential growth of the tumour, expressed as

Exp MM $$\frac{{\rm{d}}C}{{\rm{d}}t}=\mu \,C\quad C(t)={C}_{0}{{\rm{e}}}^{\mu t}.$$

The Rich MM has 4 unknown quantities (*κ*, *μ*, *α*, *C*_0_) to be estimated, the Logis MM, Gomp MM and ExpCap MM have 3 (*κ*, *μ*, *C*_0_), and the Exp MM has 2 (*μ*, *C*_0_).

### Parameter estimation

For each MM variant considered, the MM parameter likelihood function (${\mathcal{L}}$), given by Eqns. (2) or (3), and its associated Posterior, Eqn. (1), are estimated using the Markov chain Monte Carlo (MCMC) method implemented by phymcmc^17^, a graphing and analysis wrapper for emcee^18^. Specifically, emcee’s default sampler, the affine-invariant “stretch move” sampler proposed by Goodman & Weare^19^ with a stretch scale of 2^18^, is used. The initial position for each chain is log-normally distributed at random around a roughly SSR-minimised parameter set for each MM (obtained via steepest-descent).

The profile log-likelihood curves (maximum $\ln [{\mathcal{L}}]$ versus a single MM parameter) correspond to the maximum achievable ${\mathcal{L}}$ for a fixed value of one of the MM parameters while allowing all others to vary so as to maximise ${\mathcal{L}}$. The value of this maximum likelihood for one fixed parameter, and the corresponding values of the remaining parameters, is sought by using the MCMC method described above, with 40 chains of 10,000 steps. Here, no particular attention is paid to discarding burn-in steps to remove residual effects from the initial positions, to assess the independence of the parameter sets retained by the MCMC process, or to ensure the chains’ convergence. This is because the process only aims to identify and retain a single parameter set: that which yields the highest ${\mathcal{L}}$, along with its ${\mathcal{L}}$ value, out of the 400,000 parameter sets thus obtained. This procedure is repeated as the value of the fixed MM parameter is varied in small increments, shown as dots on the profile log-likelihood curves, over the interval of interest for that parameter. This approach provides only an approximation of these values, but the smooth appearance of the profile log-likelihood curves suggests sufficient accuracy for the purposes herein.

The estimated posterior likelihood distributions and associated measures correspond to all parameter sets obtained from 300 chains of 10,000 steps each, yielding 3,000,000 parameter sets, which is preceded by a burn-in of no less than 10,000 steps. The 300 chains’ initial position is selected as explained above, but chains rapidly move away from their initial position such that the effect of the latter is no longer visible after the burn-in steps are discarded. The process yields 3 million accepted parameter sets, of which at least 30,000 (1%) are completely independent based on the computed autocorrelation time^18^, which gives a rough approximation of how many MCMC steps must separate a chain’s past and present positions (parameter values) in order for the two positions to no longer be correlated.

The reported 95% credible intervals (95% CI) correspond to the Bayesian CI provided by phymcmc’s phymcmc_parstat script. The script computes the one-dimensional posterior distribution for the parameter (or the ${\log }_{10}$ of the parameter, as specified below), marginalised over all other parameters (hereafter the marginal posterior distribution, MPD), and the 95% CI bounds then correspond to the narrowest contiguous span of that parameter that encloses 95% of the MPD’s probability. This procedure can lead to strange 95% CIs when the MPD is multi-modal and the highest density 95% CI would otherwise correspond to two or more disjoint regions. For example, this is possibly an issue for *κ* in the ExpCap MM when using a linearly uniform prior.

A difference is said to be statistically significant herein if the 95% CI of one measure excludes the mean or mode (as specified) of the other measure, or if the two measures’ 95% CI do not overlap.

## Results

### Important considerations in parameter estimation

The experimental data considered herein corresponds to the control group in data published by and described in Benzekry et al.^20^. Briefly, ten C57BL6 mice were injected subcutaneously, on the caudal half of their back, with 10^6^ Lewis Lung Carcinoma (LLC) cells, said to correspond to a tumour volume of ~ 1 mm^3^. Measurements were taken by callipers and recorded in mm^3^ at various times post-injection. Due to the small tumour volumes shortly after injection, only 2/10 mice could be measured at 5 days post-injection (dpi), 8/10 at 6 dpi and 7 dpi, and 10/10 from then on. At later times, mice were euthanized for ethical reasons once tumours reached a maximum volume of 1.5 cm^3^ such that 9/10 remained at 18 dpi, 7/10 at 19 dpi, 5/10 at 20 dpi, 2/10 at 21 dpi, and only 1/10 remained at 22 dpi.

We used a Markov chain Monte Carlo (MCMC) method to sample and ultimately estimate the posterior likelihood distribution (hereafter Posterior) of each MM’s parameters from Bayes’ theorem. The Posterior of the MM parameter set $\overrightarrow{p}$, given the experimental data, is given by

1 $${\rm{Posterior}}(\overrightarrow{p}| {\rm{data}})=\frac{{\mathcal{L}}({\rm{data}}| \overrightarrow{p})\cdot {\rm{Prior}}(\overrightarrow{p})}{{\mathcal{P}}({\rm{data}})}\propto {\mathcal{L}}({\rm{data}}| \overrightarrow{p})\cdot {\rm{Prior}}(\overrightarrow{p})$$

where ${\mathcal{L}}({\rm{data}}| \overrightarrow{p})$ is the likelihood function which describes the probability of having observed the data given $\overrightarrow{p},{\rm{Prior}}(\overrightarrow{p})$ is the prior distribution for $\overrightarrow{p}$ which includes physical constraints (e.g., cannot physically be negative) and any prior knowledge (e.g., from previous measurements), and ${\mathcal{P}}({\rm{data}})$ is a normalisation factor sometimes called the evidence or the average likelihood of the data. Since we only concern ourselves with comparing the relative likelihood of the data for different $\overrightarrow{p}$, we do not need to compute the absolute likelihood and therefore can safely omit ${\mathcal{P}}({\rm{data}})$. For the likelihood of the data given $\overrightarrow{p}$, we first consider simply

2 $$\begin{array}{l}{\mathcal{L}}({\rm{data}}| \overrightarrow{p})=\exp \left[-\frac{{\rm{SSR}}(\overrightarrow{p})}{2\,{\sigma }_{C}^{2}}\right]\\\qquad\qquad\quad=\,\exp \left[-\frac{\mathop{\sum }\nolimits_{k = 1}^{{N}_{t}}\mathop{\sum }\nolimits_{{\rm{mouse}} = 1}^{\le 10}{\left\{{\log }_{10}[{C}_{{\rm{MM}}}(\overrightarrow{p},{t}_{k})]-{\log }_{10}[{C}_{{\rm{mouse}}}({t}_{k})]\right\}}^{2}}{2\,{\sigma }_{C}^{2}}\right],\end{array}$$

where SSR is the sum of squared residuals between the ${\log }_{10}$ MM-predicted tumour volume at the *k*^th^ measurement time *t*_*k*_ given $\overrightarrow{p},{C}_{{\rm{MM}}}(\overrightarrow{p},{t}_{k})$, and that observed in each mouse for which that time point was measurable, *C*_mouse_(*t*_*k*_), and ${\sigma }_{C}^{2}$ is the variance of the ${\log }_{10}$ tumour volume measurements, which is a fixed value for all mice and measurement times, as explained below. Here, *N*_*t*_ = 13 is the number of distinct measurement times in the dataset, where ${t}_{k = [1,{N}_{t}]}=\{5,6,7,11,12,13,14,15,18,19,20,21,22\}$ dpi. Eqn. (2) corresponds to the un-normalised probability density function (PDF) for the normal distribution of ${\log }_{10}C$, rather than the PDF for the log-normal distribution of *C*, and as such does not include the log-normal PDF’s normalisation factor ∝ 1/*C*. Eqn. (2) omits the normal PDF’s normalisation factor, $1/\sqrt{2\pi {\sigma }_{C}^{2}}$, because it is a constant, and we are only interested in the relative likelihood, as explained above.

**Fig. 1:**
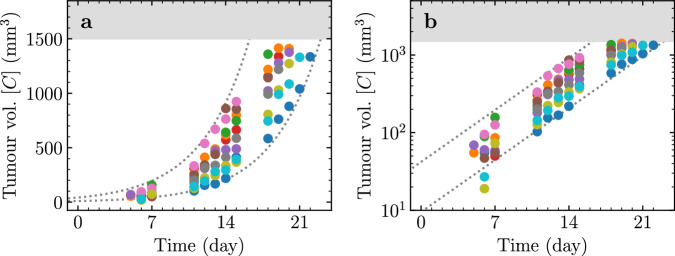
Experimentally measured tumour volume over time. The different coloured dots represent the tumour volume time course of 10 distinct mice from^20^, shown using either a (**a**) linear or (**b**) logarithmic *y*-axis. The upper grey band corresponds to the upper limit of detection (*C* = 1.5 × 10^3^ mm^3^). The dashed grey lines correspond to the Exp MM-predicted tumour volume (*C*_0_ = 20 mm^3^, *μ* = 0.22 d^−1^) multiplied or divided by 2.1 ($1{0}^{2{\sigma }_{C}}$), matching the increasing spread in measurements for larger volumes. This indicates inter-mouse variability in *C* is consistent with a log-normal distribution, following Eqn. (2).

The likelihood of the data given the parameter set was chosen to be a function of the residuals of ${\log }_{10}C$, rather than the tumour volume, *C*. This is because the inter-mouse variability in the tumour volume data is more consistent with a log-normal (${C}_{\div}^{\times}\,\text{err}$) rather than a normal (*C* ± err) distribution. This can be seen in Fig. 1 where the data is shown using both linear and logarithmic scales. The ${\log }_{10}$ tumour volume measurements appear symmetrically distributed about the mean ${\log }_{10}C$ at each time point with a constant standard deviation, i.e. one that does not depend on ${\log }_{10}C$.

Correctly choosing the weights of residuals between model and data is an important step to perform before proceeding with parameter estimation. Yet, this step is often overlooked and data variability is assumed to be normally rather than log-normally distributed. In our experience, the latter error distribution is far more commonly encountered in experimental measurements in biology^21^, notably in microbiology^12^, anatomy^22^, pharmacology^23^, neuroscience^24,25^. It follows from the central limit theorem for a process based on the multiplication, rather than the sum, of random variables. For the specific case of tumour growth, Benzekry et al.^26,27^ have demonstrated that the assumption of normal error distribution is incorrect for the measurement of growing tumour volume over time.

A fixed standard deviation *σ*_*C*_ = 0.16 was used for all data points, which corresponds to the standard deviation of ${\log }_{10}[{C}_{{\rm{mouse}}}({t}_{k})]$ across all mice at time point *t*_*k*_, averaged over all *t*_*k*_. This means that at any given time point *t*_*k*_, 95% of the ${\log }_{10}$ tumour volume measurements should fall within ± 2*σ*_*C*_ of the mean ${\log }_{10}(C)$ at that time point, or about 2.1-fold ($1{0}^{2{\sigma }_{C}}$), shown as dashed grey lines in Fig. 1.

Parameter estimation was performed using tumour volume measurements for all mice with measurable tumour volumes over all time points, rather than based on the average tumour volume at each time point. This implicitly takes into consideration the different number of measurements at each time point, avoiding the issues of having to explicitly weigh certain averaged measurement time points more or less heavily based on the number of points they represent, or to decide how best to average measurements (e.g., arithmetic vs geometric average) at each time point. This process of handling all points as a single set is mathematically equivalent to estimating the parameters’ posterior for the first mouse, subsequently using it as the prior in determining the posterior for the second mouse, and so on, a machine learning method sometimes referred to as sequential Bayesian updating.

The parameter sets explored by the MCMC runs were used not only to sample and estimate the MM parameters’ posterior, but also to efficiently sample the shape of the likelihood function around the best-fit and/or the most likely parameter set. Herein, the best-fit parameter set refers to the maximum likelihood estimate (MLE), i.e. that which maximises the likelihood function (${\mathcal{L}}$), whereas the most likely parameter set refers to the maximum a posteriori (MAP) estimate, i.e. that which maximises the joint Posterior distribution, Eqn. (1). This distinction, and specifically the Prior and Posterior distributions in Eqn. (1), will be discussed in more detail in later sections. Initially, we will focus on the likelihood function and the best-fit (MLE) parameters alone.

### Parameter values that maximise the likelihood function in each MM

**Fig. 2:**
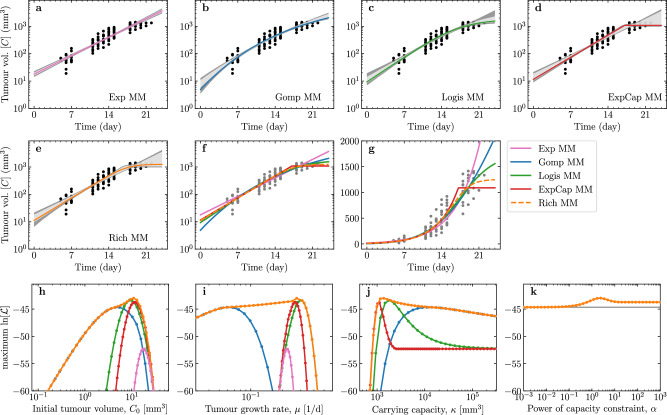
Solutions of each MM fit to the tumour volume measurements and associated parameters. **a**–**e** For each MM explored herein, the curve corresponding to the best-fit (MLE) parameter set, i.e. that which maximises the likelihood function (Eqn. (2)) is shown as a coloured line. A total of 1000 MCMC-accepted parameter sets were sampled at random with replacement to generate curves of which 68% (or 95%) fall within the pale (or dark) grey regions, respectively. **f**, **g** Tumour volume curve for each MM's best-fit (MLE) parameters against the data on a (**f**) logarithmic or (**g**) linear scale. **h**–**k** Profile log-likelihood curves for each MM parameter.

Figure 2a–g presents the solution of each MM against the data. The variability (68% and 95% credible interval) of the MM solutions is the smallest in the intermediate region when all 10 mice have measurable tumour volume, and the growth is largely purely exponential. The solutions varied most, both within each and between MMs, as the number of measurable mice decreased near the lower limit of detection at early times, and at late times when mice were euthanized before the tumour volume exceeded 1500 mm^3^.

Figure 2h–k reports the profile log-likelihood curve, i.e. the maximum $\ln [{\mathcal{L}}]$ that is attainable (Eqn. (2)) for a given value of one of the parameters, shown for each parameter in each of the MMs^4,28^. For example, given *C*_0_ = 1 mm^3^, the value of the remaining parameters is sought so as to achieve the maximum likelihood possible for this value of *C*_0_. The tumour in each mouse was initiated with the injection of 10^6^ cells, which is thought to correspond to *C*_0_ ≈ 1 mm^3^^20^. The best-fit (MLE, i.e. that which maximises Eqn. (2)) initial tumour sizes for all MMs (Fig. 2h) is larger than this estimate, namely from ~5 mm^3^ in the Gomp MM, ~10 mm^3^ in the Logis MM and Rich MM, and up to ~20 mm^3^ in the Exp MM.

In Fig. 2h, at low *C*_0_ values, the profile log-likelihood curve (max. $\ln [{\mathcal{L}}]$ vs *C*_0_) of the Rich MM corresponds to that of the Gomp MM. As *C*_0_ is increased, the maximum ${\mathcal{L}}$ improves and briefly (over a narrow range of *C*_0_ values) matches the Logis MM profile log-likelihood curve. It goes on to reach the highest ${\mathcal{L}}$ before getting worse as *C*_0_ is increased further, eventually approaching the profile log-likelihood curves of the ExpCap MM and ultimately Exp MM for the largest *C*_0_ values. Note that as different *C*_0_ values are explored (i.e., for different assumed values of *C*_0_), all other parameters are adjusting accordingly to maximise ${\mathcal{L}}$. As *C*_0_ is increased, the Rich MM profile log-likelihood curve follows that of the Gomp MM (*α* → 0), then Logis MM (*α* = 1), and finally ExpCap MM (*α* → + *∞*) curves, which suggests that larger values of *C*_0_ correspond to larger values of *α*.

Figure 2k shows that in the Rich MM, a higher likelihood is obtained for *α* = 1 Logis MM, than for *α* → 0 Gomp MM or *α* → + *∞* ExpCap MM. The best fit (MLE) is found for *α* ~ 2, but the likelihood varies far less as a function of *α*, than as a function of the other MM parameters. In particular, once *α* is smaller than ~ 0.1 (or greater than ~ 10) making *α* any smaller (or larger) neither improves nor worsens ${\mathcal{L}}$.

**Fig. 3:**
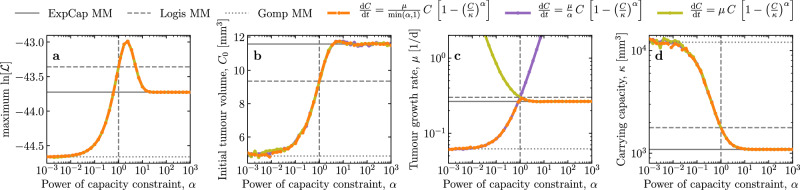
Shift in best-fit (MLE) parameters for variants of the Rich MM. Three variants of the generalised logistic growth equation are explored. The 3 coloured curves correspond to variations in the growth rate coefficient: The Rich MM as used herein with a coefficient of $\mu /\min (\alpha ,1)$ (orange dashed) is compared to expressions with a coefficient of either *μ*/*α* (purple solid) or *μ* (olive solid). The vertical dashed line indicates *α* = 1, corresponding to the Logis MM. The horizontal lines correspond to the best-fit (MLE) value of the quantity on the *y*-axis (e.g., *C*_0_, *μ*, etc.) for the ExpCap MM (*α* → + *∞*, solid), Logis MM (*α* = 1, dashed) and Gomp MM (*α* → 0, dotted). **b** The MLE value of *C*_0_ is larger as *α* gets larger. **c** While coefficient *μ*/*α* (purple) yields *μ* → + *∞* as *α* → + *∞* and coefficient *μ* (olive) yields *μ* → + *∞* as *α* → 0, the $\mu /\min (\alpha ,1)$ coefficient (orange) of the Rich MM expression used herein provides a more consistent meaning and value for parameters *μ* and *α* as *α* is varied.

Figure 3 explores the shift in the best-fit (MLE) parameters as *α* is varied in the Rich MM, and in particular the transition from the Gomp MM to the Logis MM to the ExpCap MM. The best-fit Rich MM parameters exactly match those of the Gomp MM for *α* less than about 0.1, those of the Logis MM at *α* = 1, an important transition point for the Rich MM, and those of the ExpCap MM for *α* greater than about 10. As *α* is varied from small values ≪ 1 to large values ≫ 1, the best-fit values for *C*_0_ and *κ* transition smoothly between the Gomp MM best-fit values and the ExpCap MM. There is a relatively narrow range *α* ~ (0.1, 10) where the specific value of *α* has an impact on $\ln [{\mathcal{L}}]$ and on the best-fit (MLE) values of *C*_0_ and *κ*. In Fig. 3b, the MLE of *C*_0_ vs that of *α* demonstrates, as stated above, that larger MLE values of *C*_0_ correspond to larger MLE values of *α*.

For parameter *μ*, the transition in the best-fit (MLE) value as *α* is increased is not as simple: it depends on the choice of coefficient for the generalised logistic MM. As *α* → 0, *μ* → + *∞* if the coefficient of the ODE is *μ*, or *μ* asymptotes to the Gomp MM best-fit *μ* if the coefficient is *μ*/*α*, becoming independent of *α*. For *α* → + *∞*, *μ* → + *∞* if the coefficient is *μ*/*α*, or *μ* asymptotes to the ExpCap MM best-fit *μ* if the coefficient is *μ*. This represents a change in the meaning of parameter *μ* and *α* as *α* is varied, which is problematic when trying to interpret *μ* and *α* from a biological or physical standpoint. When the coefficient is set to $\mu /\min (1,\alpha )$, as in the Rich MM, the best-fit *μ* gradually increases from its MLE value in the Gomp MM, then abruptly settles between its MLE value for the Logis MM and ExpCap MM, discontinuously rather than smoothly. This provides a more consistent physical meaning for *μ* as *α* transitions from *α* < 1 to *α* > 1. Replacing $\min (1,\alpha )$ with an expression with a smoother transition around *α* = 1, would provide a better, more gradual and consistent meaning for *μ* over the full range of the Rich MM behaviour, but we are unsure how to obtain such an expression.

**Fig. 4:**
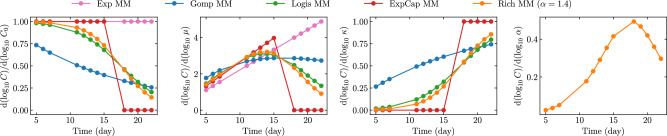
Parameter sensitivity at different measurement time points. The central difference of ${\log }_{10}C$ with respect to the ${\log }_{10}$ of each parameter about the best-fit (MLE) parameter set, computed as ${\log }_{10}[C(t,p\cdot (1+f))/C(t,p\cdot (1-f))]/{\log }_{10}[(1+f)/(1-f)]$ with *f* = 10^−8^, was used to explore the sensitivity of different measurement time points to relative, percent variations in each parameter.

Figure 4 explores how different measurement time points inform each MM parameter. This parameter sensitivity analysis relies on a local estimation of the derivative of the measurements (${\log }_{10}C$) with respect to the ${\log }_{10}$ of each parameter (relative % change) about the best-fit parameter set, following a method introduced by Miao et al.^29^. While *μ* is typically best informed by the intermediate time points, in the region where the growth is mostly exponential and all mice have measurable tumour volumes. Unsurprisingly, the initial tumour size, *C*_0_, is informed most heavily by the earliest time points, and the carrying capacity, *κ*, by the latest time points.

### Appropriately handling unmeasurable data points

In the previous section, all unmeasurable points, i.e. those for mice whose tumour was too small to be measured (12 points) and those who had to be euthanized because their tumour had reached a volume of 1500 mm^3^ (28 points), were neglected. This poses 2 problems. First, this discards meaningful information, namely that the tumour in these mice is known to be either smaller or larger than the lower or upper limit of detection, respectively. Second, it introduces a bias in the parameter estimation in that only the largest tumours measurable at early time points, and only the smallest ones at the later time points, are informing the likelihood of parameter sets. This effectively favours a theoretical curve that is higher at early times and smaller at later times than it should otherwise be.

Following the procedure described in^30^ for handling censored data, i.e. data with lower and upper limits of detection (LLD and ULD, respectively), we revise the likelihood function for the data given $\overrightarrow{p}$ as

3 $${\mathcal{L}}({\rm{data}}| \overrightarrow{p})={{\mathcal{L}}}_{{\rm{measured}}}({\rm{data}}| \overrightarrow{p})\cdot {{\mathcal{L}}}_{{\rm{unmeasured}}}({\rm{data}}| \overrightarrow{p})$$

where ${{\mathcal{L}}}_{{\rm{measured}}}({\rm{data}}| \overrightarrow{p})$ is that given in Eqn. (2), and

4 $${{\mathcal{L}}}_{{\rm{unmeasured}}}({\rm{data}}| \overrightarrow{p})=\mathop{\prod }\limits_{k=1}^{{N}_{t}}{\left[1+\frac{1}{2}{\rm{erf}}\left(\frac{{\log }_{10}[\text{LLD}/{C}_{{\rm{MM}}}(\overrightarrow{p},{t}_{k})]}{\sqrt{2{\sigma }_{C}^{2}}}\right)+\frac{1}{2}{\rm{erf}}\left(\frac{{\log }_{10}[{C}_{{\rm{MM}}}(\overrightarrow{p},{t}_{k})/\text{ULD}]}{\sqrt{2{\sigma }_{C}^{2}}}\right)\right]}^{{U}_{k}},$$

where *N*_*t*_ = 13 is as in Eqn. (2), and *U*_*k*_ is the number of mice with unmeasurable tumour volumes at time *t*_*k*_. This expression is the sum of the probability for one mouse to have tumour volume below the LLD at time *t*_*k*_, given the MM-predicted ${\log }_{10}{C}_{{\rm{MM}}}(\overrightarrow{p},{t}_{k})$,

5 $${{\mathcal{P}}}_{{\rm{LLD}}}({t}_{k})=\frac{1}{\sqrt{2\pi {\sigma }_{C}^{2}}}\mathop{\int}\nolimits_{-\infty }^{{\log }_{10}(\,\text{LLD}\,)}\exp \left[-\frac{{[x-{\log }_{10}{C}_{{\rm{MM}}}(\overrightarrow{p},{t}_{k})]}^{2}}{2{\sigma }_{C}^{2}}\right]{\rm{d}}x=\frac{1}{2}\left[1+\,\text{erf}\,\left(\frac{{\log }_{10}[\,\text{LLD}\,/{C}_{{\rm{MM}}}(\overrightarrow{p},{t}_{k})]}{\sqrt{2{\sigma }_{C}^{2}}}\right)\right],$$

and the probability for one mouse to have a tumour volume above the ULD at *t*_*k*_,

6 $${{\mathcal{P}}}_{{\rm{ULD}}}({t}_{k})=\frac{1}{\sqrt{2\pi {\sigma }_{C}^{2}}}\mathop{\int}\nolimits_{{\log }_{10}(\,\text{ULD}\,)}^{\infty }\exp \left[-\frac{{[x-{\log }_{10}{C}_{{\rm{MM}}}(\overrightarrow{p},{t}_{k})]}^{2}}{2{\sigma }_{C}^{2}}\right]{\rm{d}}x=\frac{1}{2}\left[1+\,\text{erf}\,\left(\frac{{\log }_{10}[{C}_{{\rm{MM}}}(\overrightarrow{p},{t}_{k})/\,\text{ULD}\,]}{\sqrt{2{\sigma }_{C}^{2}}}\right)\right],$$

where erf is the error function. The probability for *U*_*k*_ mice to have unmeasurable tumour volumes at *t*_*k*_, i.e. volumes that fall outside the measurable range of [LLD,ULD], is then simply ${\left[{{\mathcal{P}}}_{{\rm{LLD}}}({t}_{k})+{{\mathcal{P}}}_{{\rm{ULD}}}({t}_{k})\right]}^{{U}_{k}}$. For example, at time *t*_*k*=1_ = 5 dpi, 8 mice were unmeasurable such that *U*_*k*=1_ = 8. If all mice had measurable tumour volumes at all measurement times, i.e. if ${U}_{k}=0\,\forall \,k\in [1,{N}_{t}],{{\mathcal{L}}}_{{\rm{unmeasured}}}({\rm{data}}| \overrightarrow{p})=1$ and Eqn. (3) reduces to Eqn. (2).

While the ULD is known, namely ULD = 1500 mm^3^, the LLD will need to be estimated as an additional parameter, constrained to be ∈ (0, 19) mm^3^, where zero is excluded since some mice were unmeasurable and 19 mm^3^ is the smallest tumour volume that was measured within this data set and is therefore, at least sometimes, measurable. The fact that a 19 mm^3^ tumour can be measured does not guarantee that it is always measurable, nor that smaller tumours cannot be measured. In reality, the likelihood that a tumour volume is sufficiently large to be measured likely increases progressively for increasing tumour volumes, rather than sharply at a fixed LLD. But in the absence of sufficient data or a study intentionally designed to inform this function, a fixed LLD offers a practical alternative. Under these conditions, the most likely value for the LLD is $18.\bar{9}$ mm^3^, i.e. the closest value to the maximum allowed value for the LLD.

**Fig. 5:**
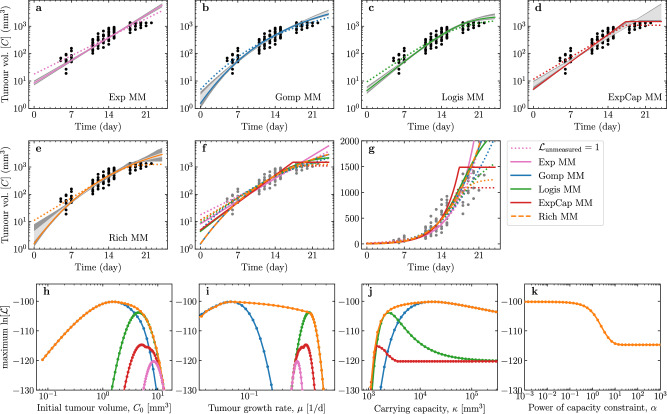
Solutions of each MM and associated parameters when including unmeasurable volumes. Panels **a**–**k** are as in Fig. 2, but correspond to the result obtained when accounting for the 38 unmeasurable tumour volumes by maximising the likelihood function defined in Eqn. (3). For comparison, the best-fit (MLE) curves when not accounting for unmeasurable data (${{\mathcal{L}}}_{{\rm{unmeasured}}}=1$, Eqn. (2)) are shown as dashed lines in panels **a**–**g**.

Figure 5 shows the new solutions and profile log-likelihood curves based on the revised likelihood function, Eqn. (3), which takes into account all 38 time points where the tumour volume was unmeasurable. The new tumour growth curves for all 5 MMs are lower at early times and higher at later times, with the old solution (dotted line) falling outside the new solution’s 95% credible interval (grey shading), when properly accounting for unmeasurable volumes. Whereas failing to account for the unmeasured tumour volumes favoured a value of *α* ~ 1.4, i.e. Logis MM-like or ExpCap MM-like rather than Gomp MM-like, accounting for these unmeasured values favours *α* → 0, i.e. Gomp MM-like growth.

Consistent with the new solutions predicting lower volumes at earlier times and higher volumes at later times, we find that the best-fit (MLE) values for the initial tumour volume, *C*_0_, are lower and those for the carrying capacity, *κ*, are higher than those of the old solution which did not account for unmeasurable volumes. The best-fit (MLE) initial tumour volume, *C*_0_, is ~1.5 mm^3^ for the Gomp MM and Rich MM, ~4.5 mm^3^ for the Logis MM and ExpCap MM, and ~8.4 mm^3^ for the Exp MM. These values are more consistent with the expected tumour volume given the number of implanted tumour cells, namely 10^6^ cells ≈ 1 mm^3^.

### Considering the impact of the parameter prior

Up until now, we have compared MMs and parameter estimation based on maximising the likelihood function alone. Now we turn our attention to ${\rm{Prior}}(\overrightarrow{p})$, an important term in Eqn. (1) which, together with ${\mathcal{L}}({\rm{data}}| \overrightarrow{p})$ discussed above, will allow us to estimate the MM parameters’ Posterior distribution.

We compare 4 possible posteriors: using the likelihood function given by Eqn. (2) or Eqn. (3), combined with either a linear or logarithmic uniform prior for all parameters. A linear uniform prior is simply ${\rm{Prior}}(\overrightarrow{p})\propto 1$, and a log-uniform prior is given by

7 $${\rm{Prior}}(\overrightarrow{p})\propto \frac{1}{{C}_{0}\,\cdot \,\mu \,\cdot \,\kappa \,\cdot \,\alpha \,\cdot \,\,\text{LLD}\,},$$

where *κ* is omitted for the Exp MM, *α* is included only for the Rich MM, and LLD is included only when the likelihood function is given by Eqn. (3), but not when it is given by Eqn. (2). The MM parameters were constrained to ${C}_{0}\in \left[0,\infty \right)\,{{\rm{mm}}}^{3},\mu \in \left[0,\infty \right)1/{\rm{d}},\kappa \in [0,1{0}^{6}]\,{{\rm{mm}}}^{3},\alpha \in (1{0}^{-5},1{0}^{5})$, and LLD = [0, 19] mm^3^. The choice of these bounds is discussed below. These constraints were imposed by setting ${\rm{Prior}}(\overrightarrow{p})=0$ when any parameter in $\overrightarrow{p}$ falls outside its bounds.

**Fig. 6:**
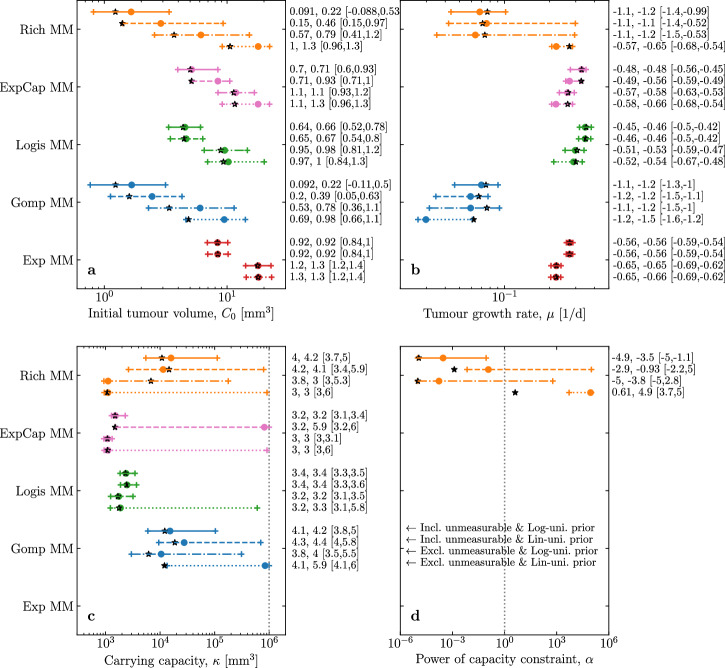
Comparing the parameter’s maxima and credible intervals under 4 different assumptions. For each parameter (different panels), for each MM (different colours) under each of the four assumptions (different line styles, annotated in the bottom right panel), the joint, multi-dimensional Posterior’s most likely parameter set (MAP, 1st number, black star), as well as the parameter’s marginal posterior distribution’s (MPD's) mode (2nd number, circle) and 95% CI (numbers in square brace, +), are provided. All numbers and CIs correspond to the probability density distributions (Posterior or MPD) for the ${\log }_{10}$ of each parameter, e.g. ${\mathcal{P}}({\log }_{10}{C}_{0})\,{\rm{d}}({\log }_{10}{C}_{0})$ rather than ${\mathcal{P}}({C}_{0})\,{\rm{d}}{C}_{0}$. The vertical dotted line corresponds to **c** the upper constraint on *κ* = 10^6^ mm^3^; or **d** *α* = 1 where the (Rich MM) corresponds to the (Logis MM).

Figure 6 shows maximum values and CIs for the ${\log }_{10}$ of each parameter, for each MM, under all 4 scenarios. Recall that herein the best-fit parameter (MLE) refers to that which maximises the joint, multi-dimensional likelihood function (${\mathcal{L}}$), and the most likely parameter (MAP) is that which maximises the joint, multi-dimensional Posterior (its mode). The joint Posterior, Eqn. (1), is not to be confused with the one-dimensional marginal posterior distribution (MPD) for a given parameter, marginalised over all other parameters, and its associated mode and 95% CI. The MAP (black star) can be markedly different from the mode of the MPD (coloured circle), especially when the data poorly constrains a parameter and the latter’s CI is very wide (e.g., Fig. 6c, d for the Rich MM parameters *κ* and *α*), and more so when a linear uniform prior is used (e.g., Fig. 6c for *κ* in the ExpCap MM). For all MMs, the MPD for the initial tumour volume (${\log }_{10}{C}_{0}$, Fig. 6a) is statistically significantly different under the assumption of a linearly uniform prior when also excluding data outside the LOD (Excl. unmeasurable & Lin-uni. prior) than under the assumption of a log-uniform prior when including data outside the LOD (Incl. unmeasurable & Log-uni. prior). The inclusion or exclusion of the unmeasurable data alone, for a given prior, was sufficient to alter ${\log }_{10}{C}_{0}$ statistically significantly (no overlap in the 2 measures’ 95% CI, see “Methods”) in the Logis MM and Exp MM. The impact is less pronounced for the tumour growth rate (*μ*), and even less so for the carrying capacity (*κ*). The mode of the joint Posterior (MLE), and the mode of the MPD of ${\log }_{10}\kappa$, both estimate a larger carrying capacity when including rather than excluding unmeasurable data, for an equivalent prior assumption. Looking at the 95% CI, however, the shift is not statistically significant (95% CIs overlap) in most cases, partly because of the CI’s width.

**Fig. 7:**
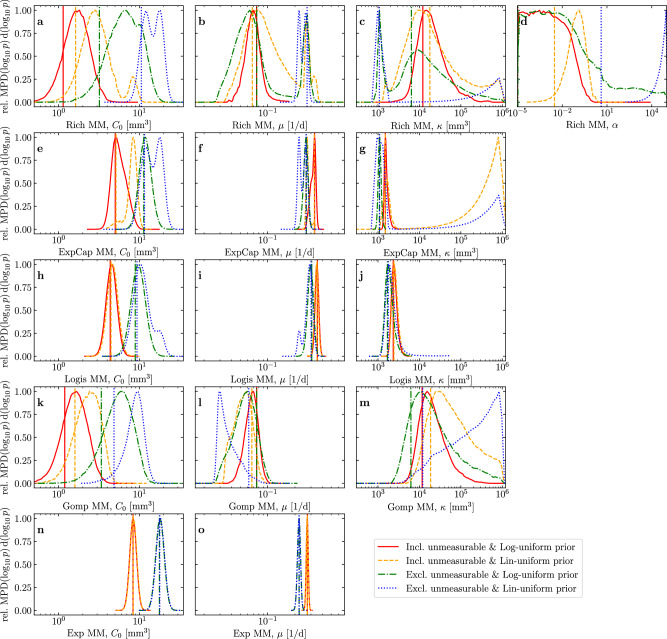
Comparing the marginal posterior distributions under 4 different assumptions. For each parameter (different columns), for each MM (different rows) under each of the four assumptions (different lines within each graph), the MAP parameter set corresponding to the mode of the joint Posterior (vertical line), as well as the MPD divided by its maximum (rel. MPD$({\log }_{10}p)\,{\rm{d}}({\log }_{10}p)$) for each parameter (curve), are provided. The Posterior and MPD are those for the ${\log }_{10}$ of the parameters, e.g. ${\mathcal{P}}({\log }_{10}{C}_{0})\,{\rm{d}}({\log }_{10}{C}_{0})$.

Figure 7 provides a more informative view of the parameters’ MPDs for each MM. In particular, it demonstrates why reporting mean or median or mode (MAP) and 95% CI can be misleading. For example, some of the MPDs for the carrying capacity (*κ*) are multi-modal, notably in Fig. 7c for the Rich MM when the unmeasurable data is excluded, and in Fig. 7g for the ExpCap MM with a linear uniform prior. These secondary modes are behind many of the widest CIs observed in Fig. 6. Such multi-modal MPDs translate poorly when reported as a 95% CI, as in Fig. 6c. This is partly because the 95% CI reported herein are computed to correspond to a single contiguous interval (see “Methods”), rather than identifying a sometimes disjoint set of bounds that would most tightly contain 95% of the MPD’s density.

A multi-modal MPD can arise out of a unimodal ${\mathcal{L}}$ when the latter is multiplied by the prior. For example, the multi-modal MPD for ${\log }_{10}\kappa$ in the ExpCap MM in Fig. 7g results from multiplying the unimodal likelihood function by the linear uniform prior. A linear uniform prior for *κ* (Prior(*κ*) d*κ* ∝ d*κ* for *κ* ∈ [0, 10^6^] mm^3^ and zero otherwise) corresponds to an exponentially increasing prior density for ${\log }_{10}\kappa$ (Prior(*y*) d*y* ∝ 10^*y*^ d*y* for *y* ∈ [− *∞*, 6] and zero otherwise, where $y\equiv {\log }_{10}\kappa$). When it multiplies the marginal ${\mathcal{L}}({\log }_{10}\kappa )$, the product results in a second mode at larger values of ${\log }_{10}\kappa$. Comparing the profile log-likelihood curves for *κ* in Fig. 5j for the ExpCap MM (red) and Logis MM (green), we see that both asymptote to a non-zero maximum ${\mathcal{L}}$ when *κ* is greater than some value, the peak and the non-zero asymptotic value of $\max ({\mathcal{L}})$ as *κ* → *∞* differ by ~ 150-fold (e^5^) in the ExpCap MM compared to ~ 10^7^-fold (e^16^) in the Logis MM. As such, the linear uniform prior was sufficient to counter the ~ 150-fold disadvantage in the ExpCap MM, but not that in the Logis MM.

The Exp MM has the narrowest posterior distributions while the Rich MM posteriors are the widest, an indication of how well the data can inform MMs with increasing numbers of parameters. As shown in Fig. 3, the shift in the maximum likelihood (MAP) as *α* is varied from 0 to + *∞* is minimal, while the best-fit (MLE) value of each parameter varies sometimes widely, e.g. *κ* varies from ~ 10^4^ to 10^3^ as *α* goes from zero to infinity. This is to be expected since *α* controls which MM the Rich MM most resembles, and all the MMs have different profile likelihood curves for each parameter. Generally, when all parameters of a MM are tightly constrained by the data, resulting in narrow profile likelihood curves, the choice of prior is less significant since the data is sufficient to overcome the effect of the prior (e.g., the Exp MM, Fig. 7n, o). Otherwise, the choice of prior can have a significant effect on the resulting posterior (e.g., *κ* in the ExpCap MM, Fig. 7g).

Priors and their bounds together should, as much as possible, correspond to all that is physically known about the parameter *prior to conducting the experiment*, no more and no less. For example, since both *C*_0_ and *κ* have the same physical dimensions as the tumour volume, and since variability in the latter is log-normally distributed, a log-uniform prior seems appropriate because (1) a uniform distribution expresses that we have no reason to prefer one set of values over another; and (2) a logarithmic scale expresses that there is an equal likelihood of finding the parameter over any one interval of ${\rm{d}}({\log }_{10}C)$, rather than d*C*, given the nature of the variability in *C* (Fig. 1). Indeed, a linearly uniform prior assumes that *κ* is 10× more likely to be found in [10,000, 20,000] mm^3^ than in [1000, 2000] mm^3^, whereas a log-uniform prior assumes that *κ* is equally likely to be found in [10,000, 20,000] mm^3^ or in [1000, 2000] mm^3^.

The more appropriate choice of prior for *μ* and *α* is less obvious: a log-uniform distribution was chosen based on our past experience applying MMs in virology, where most parameters are log-normally distributed^31–33^. Notably, the uncertainty of virus concentration measurements, like that of tumour volume measurements, is log-normally distributed^34^. Post-analysis, the profile log-likelihood curves (Fig. 5i, k) suggest these two parameters have a sensitivity (change in max ${\mathcal{L}}$ as *μ* or *α* are varied) that is symmetric on a logarithmic scale. For example, in Fig. 5k around *α* = 1, a small change, ${\rm{d}}({\log }_{10}\alpha )$ in either direction results in a similar change in max ${\mathcal{L}}$. Similarly, in Fig. 5i for the Exp MM, Gomp MM and Logis MM, the profile log-likelihood curve has a symmetric sensitivity about its mode to small changes in ${\rm{d}}({\log }_{10}\mu )$. This suggests that a log-uniform prior is probably the more appropriate choice for these two parameters, but verifying the impact of a linear versus a logarithmic uniform prior is advisable.

The prior also imposes bounds on each parameters, and these bounds should also reflect our physical knowledge. For example, we know nothing of the growth rate (*μ*), other than it should be positive, hence $\mu \in \left[0,\infty \right)1/{\rm{d}}$. Bounds on the carrying capacity (*κ*) were originally chosen $\in \left[0,\infty \right)\,{{\rm{mm}}}^{3}$. But since all values of the carrying capacity (*κ*) much larger than the largest measurable tumour volume (~10^5^ mm^3^) will make little practical difference for inference, i.e. leave ${\mathcal{L}}$ largely unchanged, an artificial upper bound of 10^6^ mm^3^ was imposed to provide a finite domain to enable the MCMC chains to converge. This, however, means that the true posterior is un-normalizable. As such, care should be taken in interpreting not only *κ*, but any other parameter whose value might correlate with *κ*, and whose MPD and MAP could shift if the artificial bound on *κ* had been handled correctly. This is also true for *α* whose value has no impact on the shape of the Rich MM-predicted tumour growth curve once it is small or large enough, i.e. beyond [10^−5^, 10^5^]. Introducing an alternative parametrization, e.g. *β* = 1 / (1 + 1 / *α*) such that $\alpha \in \left[0,\infty \right)$ maps to *β* ∈ [0, 1] with a linear uniform prior in *β*, can sometime successfully enable the full domain of the unbounded parameter to be explored by sampling and estimating the finite domain of the alternative one.

The bounds chosen for initial tumour volume (${C}_{0}\in \left[0,\infty \right)\,{{\rm{mm}}}^{3}$) deserve further discussion. Past work has often chosen to fix *C*_0_^20,26,27^, in part because the data is often insufficient to constrain all MM parameters. However, Fig. 5h demonstrates that this choice favours some MMs over others: *C*_0_ = 1 mm^3^ is well-supported (high max ${\mathcal{L}}$) by the Gomp MM, but decreasingly so for the Logis MM, ExpCap MM, and Exp MM. If we knew *C*_0_ = 1 mm^3^ to be exact, this would actually be a case of the data unambiguously rejecting some of these MMs over others. In reality, we know that *C*_0_ is not exactly equal to 1 mm^3^: the volume of 10^6^ cells would be expected to vary at least like the rest of the volume measurements as $1{0}^{0\pm {\sigma }_{{\rm{C}}}}\,{{\rm{mm}}}^{3}$, i.e. [0.47, 2.1] mm^3^. But if this initial tumour volume was measurable via callipers applied to the outside of the mouse, like the other volume measurements, it would be larger than the imagined, theoretical volume occupied by 10^6^ well-packed tumour cells, possibly by a few millimetres for each of the width and length measured to estimate the volume ($\frac{\pi }{6}\times {\text{width}}^{2}\times \text{length}\,$^26^). On the other hand, *C*_0_ could be substantially smaller than 1 mm^3^ if only a fraction of the inoculated cells ultimately form the seed from which the tumour grows, while the remaining cells are lost (e.g., phagocytosed). A more physically appropriate lower bound for *C*_0_ could be the volume of a single LLC cell, ~10^−5^ mm^3^, but it is difficult to similarly identify a suitable upper bound. Here, we decided not to constrain *C*_0_ at all, and let the data, notably the smallest measured tumour volumes, impose this upper bound. Had it been possible to physically justify setting the bounds of *C*_0_ ∈ [10^−5^, 2] mm^3^, for example, certain MMs, especially the Exp MM when excluding unmeasurable tumour volume, would have provided poor overall likelihoods, possibly disqualifying them when compared against the others.

## Discussion

In this work we explored five ordinary differential equation mechanistic models (MMs) for cancer tumour growth. We used published data^26^ of Lewis Lung Carcinoma growth in mice to constrain the parameters of the MMs which varied in complexity from 2 to 4 parameters to be estimated. We made use of Bayesian inference to estimate the MM parameters posterior distributions, and made use of the fact that the tumour volume measurement variability between mice appears to follow a log-normal distribution as the basis for our choice of likelihood function.

We demonstrated that including measurements known to be outside the upper and lower limits of detection (LOD), as opposed to simply neglecting them, has an important impact on several parameters. Notably, we showed that by discarding these data, the MM’s solutions are biased in such a way that the MM-predicted tumour volumes are higher at early times and lower at later times than when these data are included. Consequently, the best-fit (MLE) value for the initial tumour volume (*C*_0_) shifted to lower values, and that for the carrying capacity or maximum tumour size (*κ*) shifted to higher values, when properly accounting for the unmeasurable (censored) data. The value of these 2 parameters significantly alters the predicted tumour growth curves beyond the measured time points, which others have relied upon before^27^.

Many tumour growth data sets are unable to constrain all MM parameters to a satisfying extent, especially in MMs with a larger number of parameters, as was the case here for the Generalised logistic growth MM Rich MM, and to a lesser extent the Gompertz and logistic growth MMs. To address this challenge, it is common practice to fix the value of some of the MM parameters. For example, *C*_0_ is sometimes fixed based on the number of tumour cells injected into the animal. For the data set used here, this value is said to be 10^6^ cells ≈ 1 mm^3^. By estimating *C*_0_ for all our MMs, we found that the best-fit (MLE) value of *C*_0_ was the smallest for the Gompertz MM, followed by the logistic and exponential growth MMs. As such, by fixing *C*_0_ to a certain value, one favours some MMs at the expense of others, leading to MM selection bias. Since the MM choice also biases the MM-predicted tumour growth curve beyond the measured time points, this can be particularly problematic in applications where these MMs are pushed beyond the region of validation. It also means that an experimental study designed to accurately measure *C*_0_, or at least aiming to measure the smallest possible tumour volumes, would be particularly helpful in informing MM selection.

Simpson et al.^35^ used the profile log-likelihood to explore the practical identifiability of three sigmoid curves (Logis MM, Gomp MM, and a version of Rich MM) based on coral data. Their dataset included later time points indicating a slowing down of the growth, and they found, rather surprisingly, that the Logis MM model was practically identifiable whereas the Gomp MM was not, specifically for parameter *C*_0_. The Gompertz model is a very common model of tumour growth, possibly because it often recapitulates the desired dataset quite well^36^. Its limitation, however, is the non-mechanistic description of growth slowdown that occurs with increasing tumour sizes. Herein, we refrain from recommending any MMs over others, in part because the dataset used lacks measurements of sufficiently small tumours to inform early growth kinetics, and of sufficiently large tumour volumes to characterise the carrying capacity. Yet, these two regimes are where the 5 MM’s predictions differ most and, as such, are critical to robustly inform model selection. The goal of the present work was to show how often overlooked decisions in parameter estimation can impact key results and conclusions, including model selection. The process of model selection requires an accurate assessment of the goodness-of-fit and model complexity, relying on measures such as the Akaike or Bayesian information criteria, see for example^37,38^. A number of choices made herein (e.g., setting arbitrary bounds on *κ* and *α*) have biased and artificially bounded what would otherwise have been improper posteriors in some MMs, and would compromise the reliability of a model selection analysis.

We also showed that the choice of priors, and their corresponding bounds, when using Bayesian parameter estimation can have a significant effect on the resulting posterior and most likely parameter set (MAP). This effect is stronger when data is limited^39^, as is often the case in mathematical oncology applications. Truncated normal distributions^40^ or uniform distributions^41–43^ are common choices for priors. Guckenberger et al.^44^ tested the sensitivity of their posteriors to several choices of prior, including normal, Gamma, and uniform distributions. Where parametrised MMs are applied to simulate tumour growth by sampling parameters from assumed distributions, log-normal distributions are sometimes chosen^45^, because they ensure sampled parameters are positive, and the resulting simulated dataset has a low mean and a high variance^46^, such as tumour volume measurements^47^. But in the absence of any knowledge about the parameters, linear and logarithmic uniform priors appropriately reflect this lack of knowledge about the parameters, relying instead on the data to inform the posterior. Based on the present data, the profile log-likelihood curves’ sensitivity to each parameter suggests that log-uniform priors are a more appropriate choice for all parameters of the 5 tumour growth MMs explored in this work. It is unclear whether this is a property of these MMs, or is also partly a property of the data. As such, future work should continue to evaluate these two priors and their effect on the results.

In summary, we proposed an easy to re-use mathematical framework based on Bayes’ theorem to estimate MM parameters in a manner that better captures inter-individual tumour volume measurement variability, and incorporates all measurements, including those beyond the LOD. The framework provides not only a robust way to identify the best-fit (MLE) parameters, i.e. those that maximise the likelihood function but also a parameter and growth curve sensitivity analysis in the form of a distribution of solution curves and the shape of the maximum likelihood function about these parameters, i.e. the profile log-likelihood curve^4,28^. We recommend that future works suitably evaluate the impact of physically justifiable priors (linear and logarithmic uniform) on Bayesian parameter estimation in mathematical oncology applications, where data is often limited and noisy, and such choices can make a significant difference. We encourage the presentation of more complete, graphical views of parameter posterior distributions, rather than just modes and credible intervals, so these impacts can be better understood and eventually better managed.

## Data Availability

No experimental data was generated as a part of this study. All experimental data used in this study was previously published and cited accordingly in reference^20^.

## Code availability

The mathematical algorithms used in this work are freely available at https://github.com/cbeauc/phymcmc^17^.
